# Joint pre-processing framework for two-dimensional gel electrophoresis images based on nonlinear filtering, background correction and normalization techniques

**DOI:** 10.1186/s12859-020-03713-0

**Published:** 2020-08-31

**Authors:** Manuel Mauricio Goez, Maria C. Torres-Madronero, Sarah Rothlisberger, Edilson Delgado-Trejos

**Affiliations:** 1 Smart Machine and Pattern Recognition Laboratory - MIRP, Faculty of Engineering, Instituto Tecnologico Metropolitano ITM, Medellin, 050012 Colombia; 2 Biomedical Innovation and Research Group, Faculty of Applied and Exact Sciences, Instituto Tecnologico Metropolitano ITM, Medellin, 050034 Colombia; 3 AMYSOD Lab (Parque i), CM&P Research Group, Quality and Production Department, Instituto Tecnologico Metropolitano ITM, Medellin, 050034 Colombia

**Keywords:** Adaptive histogram equalization, Multilevel thresholding, Nonlinear diffusion, Pre-processing, Two-dimensional gel electrophoresis

## Abstract

**Background:**

Two-dimensional gel electrophoresis (2-DGE) is a commonly used tool for proteomic analysis. This gel-based technique separates proteins in a sample according to their isoelectric point and molecular weight. 2-DGE images often present anomalies due to the acquisition process, such as: diffuse and overlapping spots, and background noise. This study proposes a joint pre-processing framework that combines the capabilities of nonlinear filtering, background correction and image normalization techniques for pre-processing 2-DGE images. Among the most important, joint nonlinear diffusion filtering, adaptive piecewise histogram equalization and multilevel thresholding were evaluated using both synthetic data and real 2-DGE images.

**Results:**

An improvement of up to 46% in spot detection efficiency was achieved for synthetic data using the proposed framework compared to implementing a single technique of either normalization, background correction or filtering. Additionally, the proposed framework increased the detection of low abundance spots by 20% for synthetic data compared to a normalization technique, and increased the background estimation by 67% compared to a background correction technique. In terms of real data, the joint pre-processing framework reduced the false positives up to 93%.

**Conclusions:**

The proposed joint pre-processing framework outperforms results achieved with a single approach. The best structure was obtained with the ordered combination of adaptive piecewise histogram equalization for image normalization, geometric nonlinear diffusion (GNDF) for filtering, and multilevel thresholding for background correction.

## Introduction

A commonly used gel-based approach for proteomic analysis is two-dimensional gel electrophoresis (2-DGE), a technique that separates proteins in a sample based on both their isoelectric point and molecular weight [1]. This technique is often used in preliminary comparative proteomic analyses, as it is capable of resolving thousands of proteins in a single run. Once the proteins in the sample have been separated, the gel is then scanned and the imaged processed using computational tools. Often these 2-DGE images exhibit anomalies due to the technique itself or to the image scan and acquisition [2]. The purpose of 2-DGE image analysis is to detect the proteins (black spots) within the gel. However, a noisy background with variable intensity, diffuse or low-intensity spots, and over-saturated spots often hinder the detection of individual proteins. Therefore, a pre-processing step that minimizes these anomalies is an open issue in the literature, as an important phase prior to analysis of these kinds of images [3].

Pre-processing techniques for 2-DGE image analysis are classified as: image normalization, background correction, and noise reduction techniques [3, 4]. Image normalization improves the detection of low abundance proteins (low-intensity spots) [5]. Satisfactory image normalization results are achieved using multiple gels, obtaining a pattern that is compared with each sample; however, aligning the multiple images is the main difficulty of this technique [6]. On the other hand, the aim of background correction is to increase contrast and decrease the effects of non-homogeneous regions, thus improving spot detection. In the literature, there are several background correction techniques reported for 2-DGE image processing, such as adjustment by either local or global minima, polynomial adjustment, and approaches based on histograms [6, 7]. Despite the advances in normalization and background correction techniques, noise reduction approaches have been the most studied for 2-DGE image pre-processing. We found several linear and nonlinear filters used for noise reduction of 2-DGE images [3, 4]. Usually, linear filters blur the spots and reduce their intensities, which is not optimal as it alters the end results [8]. Thus, it is common to use nonlinear filters, such as filters based on Wavelet [3], Contourlet [9] and total variation (TV) [10]. The most commonly used nonlinear filtering technique for 2-DGE is based on Wavelet transform, which achieves high noise reduction; however, with this technique it is difficult to preserve the spot contours [3, 4]. On the other hand, TV preserves better spot edges due to a smoothing variable operation, but is limited in terms of noise reduction [10]. Contourlet transform also performs better than Wavelet in preserving edge information [9]. Xin and Zhao [11] used a combined version of Wavelet and TV (WTTV) to reduce information loss in 2-DGE image pre-processing. In a previous work [4], we presented a comparison between Wavelet, Contourlet, TV, and WTTV filters using synthetic and real 2-DGE images, showing that with synthetic data, Wavelet and WTTV had the lowest sensitivity to noise levels, while wavelet presented the best detection rate for known proteins on real 2-DGE images. However, these results were obtained by executing each technique separately and a joint framework was not considered.

Noise reduction, image normalization and background correction techniques reduce specific anomalies in 2-DGE images. For example, noise reduction minimizes the effect of impulsive and white noise; image normalization normalizes over-saturated and low abundance spots, as well as light saturation; and background correction reduces variability, saturation and streaking. Since each approach reduces a specific anomaly in 2-DGE images, it is necessary to combine them in order to enhance the spots in the image. This paper discusses a joint framework that combines the capabilities of image normalization, background correction and nonlinear filtering. Since there are several techniques for each approach, we first present a comparative study using both synthetic and real 2-DGE images and then we evaluate the combined framework. For this comparison, we used four metrics to evaluate the performance of the techniques applied to synthetic data, and we evaluated their capabilities in reducing anomalies in real 2-DGE images.

## Pre-processing framework for 2-DGE images

In the proposed framework, the first step is image normalization. This step improves the contrast of protein spots, mainly low intensity ones. As in the literature there are several normalization techniques, we compared three enhancement techniques: histogram equalization, adaptive piece-wise histogram equalization [12], and a modification of background pixel intensity [7].

As mentioned previously, image normalization improves the contrast of low intensity protein spots; however, it also increases both the intensity of isolated points and impulsive noise. Therefore, in the proposed joint pre-processing framework, noise reduction is the second step in the process. For noise reduction, nonlinear filtering techniques are recommended for low edge distortion. A comparison of the most commonly used nonlinear techniques for 2-DGE image is presented in [4]. Quantitative comparison showed that Wavelet filtering performs better than Counterlet, TV, and WTTV. However, the results in [4] showed that with Wavelet there was less noise reduction but edge information was better preserved than with other techniques. In this paper, we evaluate the use of geometric nonlinear diffusion filtering (GNDF) for the pre-processing of 2-DGE images [13].

Finally, background correction techniques achieve better results when processing images with low levels of noise, therefore it is the last step in the pre-processing framework. We compared thresholding, multilevel thresholding [7] and surface approximation [14].

### Image normalization

The histogram is an estimation of the probability of occurrence of grey levels in an image. The histogram is given by [15]:

1 $$ p(k)=\frac{n_{k}}{n}\quad k=0,1,\ldots,L-1  $$

where *n* is the total number of pixels in the image, *n*_*k*_ is the number of pixels with grey levels equal to *k*, *L* is the number of possible grey levels, and *p*(*k*) is the probability of occurrence of *k*. Histogram equalization is an image transformation that approaches the probability of occurrence of grey levels to a uniform probability density function. This transformation improves the use of the dynamic range for grey levels, thus improving contrast. From the histogram, the histogram equalization is obtained by computing the function *S*_*k*_ given by:

2 $$ s(k)=\sum_{j=0}^{k}\frac{n_{k}}{n}\quad k=0,1,\ldots,L-1  $$

and then mapping each pixel with level *k* in the equalized image with a pixel value equal to ⌊(*L*−1)*S*_*k*_⌋.

Given that pixel intensities behave randomly due to the type of sample and the acquisition process, an adaptive piecewise histogram equalization is proposed in [12]. This technique performs multiple histogram equalizations considering the maximum and minimum intensity levels. Further details of the algorithm are in [12].

Another way to perform image normalization is to modify the background pixel intensity [7]. The background of the image is estimated using a threshold and then it is subtracted from the data.

### Nonlinear filtering

GNDF [13] reduces noise while preserving edge information, so it is expected to improve spot detection in 2-DGE image analysis. GNDF solves a nonlinear differential partial equation given by:

3 $$ \frac{\partial I}{\partial t}=\frac{d}{dx}[C|\nabla I|*\nabla I]  $$

where the initial condition *I*(*t*=0) is the 2-DGE image, ∇*I* is the image gradient, and *C* are the diffusion coefficients defined as:

4 $$ C(x)=\frac{1}{1+(x/k)^{2}}  $$

where *k* is a threshold that determines the level of noise to be removed. The estimation of *k* is obtained from the signal to noise ratio of the image [13].

In addition to GNDF, in this study we used Wavelet Transform for noise reduction. A comparison of WT and other filtering techniques is presented in [4]. We use WT with a Daubechies family and 5 levels of decomposition [2, 4].

### Background correction

We compared three background correction techniques: thresholding, multilevel thresholding [7] and surface approximation [14]. Thresholding estimates the intensities of background pixels to be subtracted from the image. Since most of the time the background of 2-DGE images is not homogeneous, techniques such as multilevel thresholding can yield better results. Multilevel thresholding divides the image into several regions, and in each region we can estimate the intensities of the background pixels. For this paper, two levels *G*_*f*1_ and *G*_*f*2_ are used:

5 $$\begin{array}{@{}rcl@{}} G_{f1} = I \in \left(I_{i}< \frac{G_{1}}{n_{1}} \right) \quad &\text{where}& \quad G_{1}= \sum P_{x}(0, \widetilde{P_{x}}) \end{array} $$

6 $$\begin{array}{@{}rcl@{}} G_{f2} = I \in \left(\frac{G_{1}}{n_{1}} < I_{i}< \frac{G_{2}}{n_{2}} \right) \quad &\text{where}& \quad G_{2}= \sum P_{x}(\widetilde{P_{x}}, maxP_{x}) \end{array} $$

where *G*_*f*1_ is the first level, with pixels of intensities between the minimum grey level and the median of a percentile *P*_*x*_ ($\widetilde {P_{x}}$), and *G*_*f*2_ is the second level with pixels of intensities between $\widetilde {P_{x}}$ and the maximum value of the percentile *maxP*_*x*_.

A third method used in this paper for background correction is surface approximation [7]. A B-Spline surface is used to estimate background with the iterative algorithm presented in [7].

## Experiments

### Databases

#### Database 1: synthetic dataset

Synthetic proteins were modelled as two-dimensional Gaussian distributions [16], assuming the media, *μ*, and standard deviation, *σ*, are equal for both dimensions. Size and scattering for a protein are varied through *σ*. Protein location within a synthetic image was randomly generated using a uniform distribution. The random distribution generated some overlapping spots. Gaussian, Rayleigh and exponential noise, given by (7), (8) and (9) respectively, were added to the synthetic images. The parameters presented in [4] were used for each noise in order to simulate images with signal-to-noise ratio -SNR between 8 and 20 *db*.

7 $$\begin{array}{@{}rcl@{}} p(z) &=& \frac{1}{ \sqrt{2 \pi \sigma} } \exp^{-(z-\mu)^{2}/ 2 \sigma^{2}}  \end{array} $$

8 $$\begin{array}{@{}rcl@{}} p(z) &=& \frac{2}{b}(z-a)\exp^{-(z-a)^{2}/ b}  \end{array} $$

9 $$\begin{array}{@{}rcl@{}} p(z) &=& a \exp^{-az}  \end{array} $$

#### Database 2: ITM 2-DGE image database

This dataset was collected from previous studies carried out in the Laboratory of Molecular and Cell Biology of the Instituto Tecnologico Metropolitano ITM of Medellin (Colombia). The 2-DGE images correspond to two different sample types:

- Bee venom collected from africanized worker bees (samp_01–02–03 and 04).
- Urine samples taken from patients with prostate cancer (samp_05 and 06).

Proteins (50 *μg*) were loaded by passive re-hydration onto 7 *cm* ZOOM ^®^ IPG (Immobilized pH gradient) strips pH 3-10 NL (ThermoFisher Scientific). Isoelectric focusing was carried out using the following voltage ramp: 200−450−600−750−950 *V* during 25 *min*, 1200−1400−1600 *V* during 30 *min*, and 2000 *V* during 45 *min* [17]. For the second dimension, the IPG strips were loaded onto SDS-PAGE NuPAGE™ 4−12*%* Bis-Tris Protein Gels, 1.5 *mm* (ThermoFisher Scientific) and run at 200 *V* during 40 *min*. After electrophoresis these were stained with SYPRO™ Ruby (Invitrogen™, ThermoFisher Scientific) and the gel images were acquired using the ChemiDoc MP System (Bio-Rad). Gel images were analyzed and compared using the PDQuest Advanced 2-D Software (Bio-Rad).

#### Database 3: lECB 2-D PAGE gel image database

This database consist of four 2-DGE image data sets previously analyzed with the GELLAB-II system [18]. These data sets consist of over 300 gel images (gif format) with annotations and landmark data in html, tab-delimited and xml formats. The data sets and experimental conditions are described and documented in the papers associated with each data set [19–22]. From this database, four 2-DGE images were randomly selected for this study, one from each data set:

1. Human leukemias/gel-HM-029 (samp_07)
2. HL-60 cell line/gel-HL60-HUM-MYEL-DIFF-029 (samp_08)
3. MOLT-4 cell line/gel-MOLT-4-004 (samp_09)
4. Fetal Alcohol Syndrome (FAS) - serum/gel-FAS-NA-NA-001 (samp_10)

This database is available for public use and can be downloaded from

http://www.bioinformatics.org/lecb2dgeldb/.

### Validation measures

In this study four indicators were used for evaluating the performance of pre-processing techniques. For evaluating normalization, we used the percentage of low-abundance proteins detected (LPD) defined as the ratio between the number of low-abundance spots detected (LAS _*det*_) and the total number of low-abundance spots (LAS _*tot*_) in the image:

10 $$  \text{LPD} = \frac{\text{LAS}_{det}}{\text{LAS}_{tot}}  $$

In the case of noise reduction techniques, the signal to noise ratio (SNR), based on the normalized mean square error (MSE _*n*_), was used and can be given by:

11 $$\begin{array}{@{}rcl@{}} \text{MSE}_{n} &=& \frac{\sum_{i=1}^{n}(x_{i}- \widehat{x}_{i})^{2}}{\sum_{i=1}^{n}(x_{i})^{2}} \end{array} $$

12 $$\begin{array}{@{}rcl@{}}  \text{SNR} &=& 10*\log_{10}\frac{1}{\text{MSE}_{n}} \end{array} $$

where *x*_*i*_ is a pixel in the original image and $\widehat {x}_{i}$ is the same pixel in the filtered image. Additionally, spot efficiency (*Ξ*) was used to evaluate the performance of noise reduction techniques, in terms of the number of true detected spots (*ς*_*t*_), false detected spots (*ς*_*f*_) and lost spots (*ς*_*l*_) [3, 4]:

13 $$  \Xi = \frac{\varsigma_{t} - \varsigma_{f}}{\varsigma_{t} + \varsigma_{l}}  $$

Finally, the background correction methods were evaluated using the background subtraction index (BSI), which was calculated in terms of the number of detected pixels that belong to the background (*ϱ*_*det*_) and the total number of pixels that belong to the background (*ϱ*_*tot*_). Thus, BSI means the percentage of pixels identified as background:

14 $$ \text{BSI} = \frac{\varrho_{det}}{\varrho_{tot}}  $$

### Proposed approach

According to the measures expressed by (10), (12), (13) and (14), several configurations of stages for normalization, noise reduction and background correction were tested in a sequential structure made up by three stages, named in this work as the joint pre-processing framework. In this sense, the order of the stages was an important aspect to evaluate and the performance of several techniques in each stage was registered, in order to find the most effective structure configuration, which was validated by experts. It is important to note that the training was executed using synthetic images, but the validation was performed using real 2-DGE images, where the algorithm results were compared with the expert’s opinions.

## Results and discussion

### Comparison of normalization techniques

**Fig. 1:**
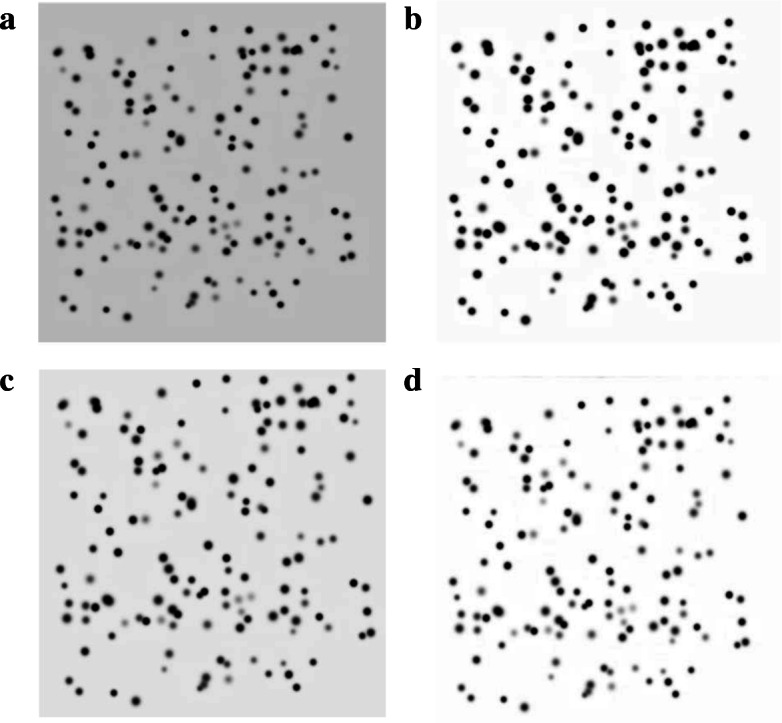
Synthetic protein spots modelled as a 2-D Gaussian distribution. **a** Example of a synthetic image. **b** Synthetic image normalized using histogram equalization. **c** Synthetic image normalized using adaptive piecewise histogram equalization. **d** Synthetic image normalized using modification of background pixel intensity

As image normalization seeks to enhance low-abundance proteins, we used a synthetic image with these kinds of spots (see Fig. 1a). The synthetic image had 1024 x 1024 pixels, with an opaque background and 150 spots, which were generated by a Gaussian distribution with standard deviation between 0.3 and 0.8. The spot intensity was controlled to simulate low-abundance proteins with a grey level between 0.1 and 0.8. We compared histogram equalization, adaptive piecewise histogram equalization [12], and a modification of background pixel intensity [7] for image normalization, and used the percentage of low-abundance proteins detected (LPD) to evaluate the performance of each technique.

The LPD results are presented in Table 1. The technique based on background pixel intensity detected only 48.7% of low-abundance spots. On the other hand, the histogram and adaptive piecewise histogram equalizations detected 82.1% and 88.9% of low abundance spots, respectively. As can be seen in Fig. 1b and c, the techniques based on equalization enhanced the contrast of the low-abundance spots.

**Table 1:** Performance of image normalization techniques for a synthetic image with low-abundance spots evaluated using LPD

Technique	LPD(%)
Background pixel intensity	48.7
Histogram equalization	82.1
Piecewise equalization	**88.9**

The values in bold indicate the best LPD achieved.

**Fig. 2:**
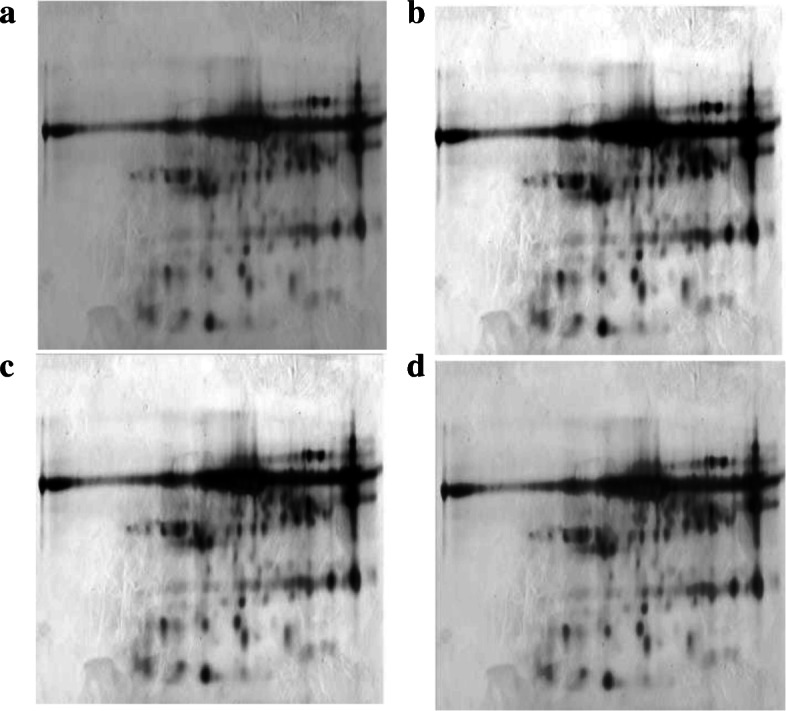
Two-dimensional gel electrophoresis – 2-DGE – image from a human urine sample (samp_05). **a** Original image. **b** 2-DGE image normalized using histogram equalization. **c** 2-DGE image normalized using adaptive piecewise histogram equalization. **d** 2-DGE image normalized using modification of background pixel intensity

Figure 2 presents the normalization results for a real 2-DGE image (samp_05). The equalization-based approach improves contrast by increasing the grey level intensity of the protein spots and decreasing the intensity of the background pixels (see Fig. 2b and c). However, normalization also increases the background noise, so it was necessary to combine image normalization with a noise reduction technique.

### Comparison of noise reduction techniques

Wavelet transform (WT) is one of the nonlinear filters that presents the best performance for noise reduction in 2-DGE images [4]. However, there are other nonlinear methods that allow noise reduction without smoothing spot edges. We compared WT with geometric nonlinear diffusion filtering - GNDF. GNDF has been shown to perform well with several types of medical images but has not been used with 2-DGE images. For WT filter, a Daubechies wavelet family was used with five decomposition levels [4]. For GNDF, we used 35 smoothing iterations with a diffusion coefficient equal to 0.2 and windows of 5x5 pixels. The performance was evaluated using the signal-to-noise ratio (SNR) and spot efficiency [4]. WT and GNDF were tested with synthetic images with Gaussian, Rayleigh and exponential noise with SNR from 20 to 8 dB. Each synthetic image has 512x512 pixels with 250 spots.

Table 2 presents the spot efficiency comparison using WT and GNDF filters for the synthetic images with noise. In terms of spot efficiency, WT and GNDF yielded very similar results for most noise levels, with differences close to 2%. However, for the synthetic image with Gaussian noise of 8 dB (i.e. the higher noise level), GNDF presented a spot efficiency of 77.86%, while WT obtained 67.5%. On the other hand, better results were obtained by GNDF in terms of SNR. Table 3 shows the SNR comparison for WT and GNDF filters. In the case of the image with SNR of 8dB, WT obtained images with 19.31 dB, 9.78 dB and 12.71 dB for the Gaussian, Rayleigh and exponential noise respectively; while GNDF obtained images with 20.11 dB, 10.5 dB and 15.61 dB for Gaussian, Rayleigh and exponential noise respectively.

**Table 2:** Performance of noise reduction techniques evaluated using spot efficiency (%)

Noise type	Noise reduction technique	Noise intensity (dB)						
		20	18	16	14	12	10	8
Gaussian	WT	**90.36**	**90.71**	89.29	**89.29**	**88.93**	85.00	67.50
	GNDF	88.57	88.21	**89.64**	88.57	84.29	**85.36**	**77.86**
Rayleigh	WT	90.00	**90.36**	**90.71**	**89.64**	**89.29**	**87.14**	**90.00**
	GNDF	**90.71**	88.93	89.29	87.86	88.21	86.43	87.14
Exponential	WT	**91.07**	91.43	90.71	90.36	**89.29**	84.29	82.86
	GNDF	90.71	**89.64**	89.29	**90.71**	88.57	**87.86**	**82.87**

The values in bold indicate the best spot efficiency for each noise level.

**Table 3:** Performance of noise reduction techniques evaluated using SNR (dB)

Noise type	Noise reduction technique	Noise intensity (dB)						
		20	18	16	14	12	10	8
Gaussian	WT	27.60	26.69	25.63	24.30	22.83	21.15	19.31
	GNDF	**28.42**	**27.30**	**26.09**	**24.89**	**22.98**	**21.87**	**20.11**
Rayleigh	WT	27.60	24.76	20.99	17.80	14.94	12.34	9.78
	GNDF	**28.40**	**25.69**	**21.77**	**18.47**	**15.52**	**12.89**	**10.50**
Exponential	WT	**25.08**	26.62	24.73	21.48	18.27	15.39	12.71
	GNDF	24.67	**26.88**	**26.79**	**24.61**	**21.55**	**18.49**	**15.61**

The values in bold indicate the best SNR for each noise level.

**Fig. 3:**
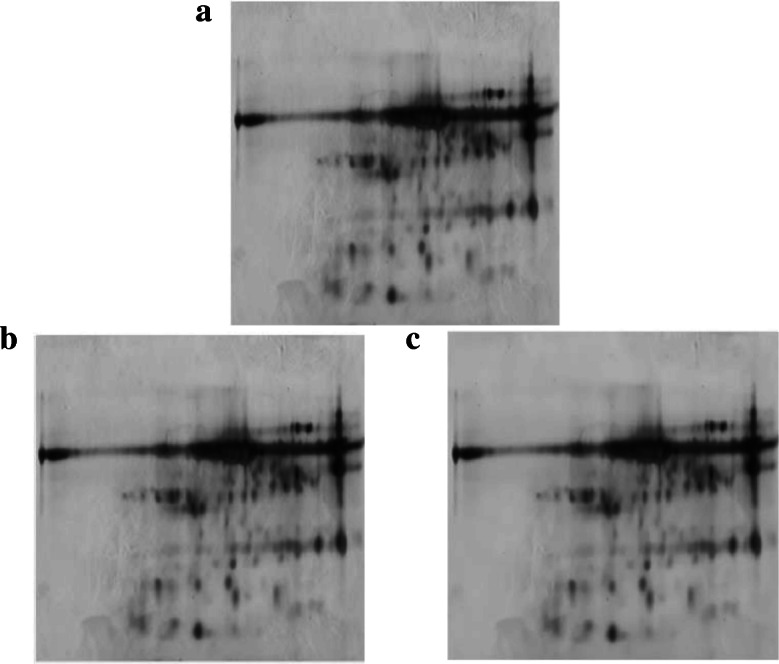
Two-dimensional gel electrophoresis – 2-DGE – image from a human urine sample (samp_05). **a** Original image. **b** 2-DGE image filtered using Wavelet Transform -WT. **c** 2-DGE image filtered using geometric nonlinear diffusion filtering - GNDF

Both nonlinear filtering techniques, WT and GNDF, were applied to real 2-DGE images (samp_05). As can be seen in the results in Fig. 3, the effect of filtering can be noted in the background, as GNDF reduces the background noise while preserving the spot contours.

### Comparison of background correction

**Fig. 4:**
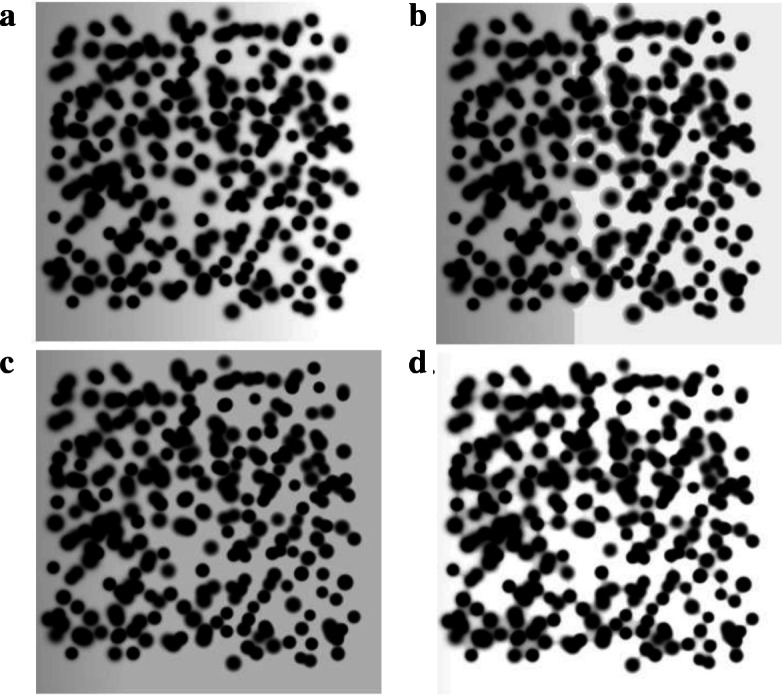
Synthetic protein spots modelled as a 2-D Gaussian distribution with background. **a** Example of a synthetic image. **b** Synthetic image with background correction using thresholding. **c** Synthetic image with background correction using multilevel thresholding. **d** Synthetic image with background correction using surface approximation

We compared three background correction techniques: thresholding, multilevel thresholding [7] and surface approximation [14]. First, we generated a synthetic image with changes in background intensity (see Fig. 4a). The background variation was obtained by increasing the initial intensity up to 155%. A percentile of 60% was used for both thresholding techniques. A B-Spline equation [14] was used for the surface approximation techniques optimizing the parameters with 150 iterations. The performance was evaluated by the Subtraction Index (SI) that compares the number of background pixels with the estimated.

Figure 4 presents the background correction results in the synthetic image. Using thresholding, the background was partially removed, but as can be seen in part B of the figure, the background is divided in two regions. Conversely, a uniform background was obtained with multilevel thresholding. The surface approximation removed most of the background, but this technique did not work for pixels close to the spots. The SI results are presented in Table 4. Thresholding detected 71.8% of background pixels, while surface approximation and multi-level thresholding detected 97.9% and 98.5% of background pixels for the synthetic images respectively.

**Table 4:** Performance of background correction techniques for a synthetic image with variable background using BSI

Technique	BSI(%)
Histogram-based	71.8
Modified histogram-based	**98.5**
Surface approximation	97.9

The values in bold indicate the best BSI achieved.

**Fig. 5:**
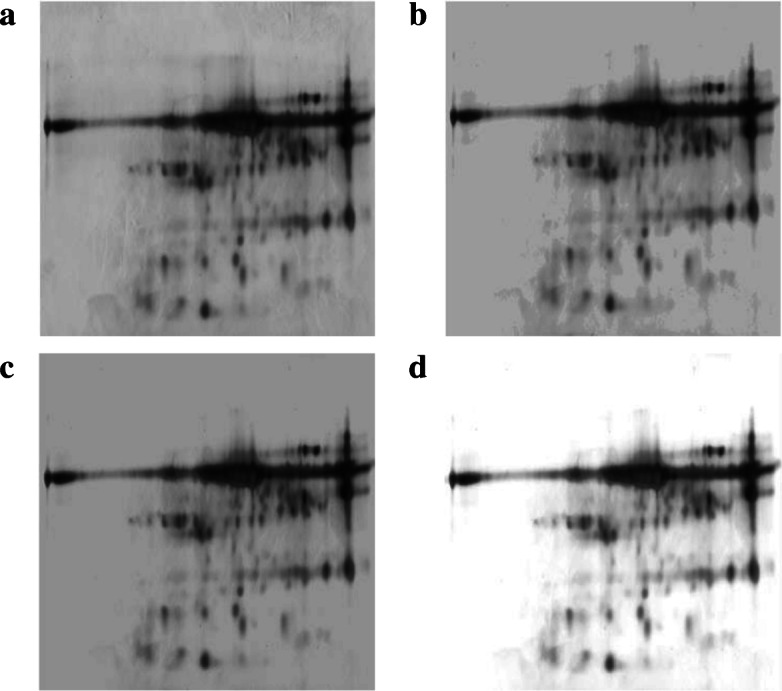
Two-dimensional gel electrophoresis – 2-DGE – image from a human urine sample (samp_05). **a** Original image. **b** 2-DGE image with background correction using thresholding. **c** 2-DGE image with background correction using multilevel thresholding. **d** 2-DGE image with background correction using surface approximation

Figure 5 presents the background correction for a real 2-DGE image (samp_05). Thresholding preserved background intensities around spots, but the background obtained from multi-level thresholding and surface approximation approaches was uniform and increased spot contrast. However, background noise was also preserved; hence, it is necessary to combine background correction with noise reduction techniques for pre-processing of 2-DGE images.

### Proposal novelties I: joint pre-processing framework

Based on the comparison of image normalization, noise reduction and background correction techniques, we show that a joint pre-processing framework is needed. The proposed framework takes advantage of the capabilities of image normalization to increase the contrast of low-abundance proteins, of nonlinear filtering to reduce noise while preserving edge information, and of background correction to homogenize background pixels. According to previous results, we used piecewise histogram equalization for image normalization, GNDF for filtering and multi-level thresholding for background correction. The joint pre-processing framework was evaluated using both synthetic and real 2-DGE images.

The joint pre-processing framework was evaluated using a synthetic image generated by a 2-D Gaussian distribution, where the 150 spots have a standard deviation between 0.1 and 0.8. The image includes an intensity variation in the background along the horizontal axis. Additionally, the image has Gaussian noise with a median of zero, standard deviation equal to 1.535 and Rayleigh noise with a = 0 and b = 0.0539. Table 5 presents the performance results using LPD, spot efficiency and SI. The SI metric was only computed for the images obtained from the background correction and joint pre-processing techniques, as it measures the background subtracted from the image.

**Table 5:** Performance of the joint pre-processing framework for a synthetic image with variable background and noise using LPD, spot efficiency (*Ξ*), and BSI

Technique	LPD(%)	*Ξ* (%)	BSI(%)
Original synthetic image	0	5.87	N.A
Piece wise equalization	40	3.57	N.A
GNDF	3.33	17.69	N.A
Modified histogram-based	0	6.69	11.37
Joint pre-processing framework	**60**	**63.84**	**78.62**

The values in bold indicate the best LPD, spot efficiency, and BSI achieved.

The best LPD was obtained using the joint pre-processing framework with 60% of low-abundance spots detected in the image. By comparison, this percentage was 40% when only the normalization technique was implemented. In terms of spot efficiency, the proposed framework detected 63.84% of spots, while lower percentages were obtained when using a single technique: 3.57% for normalization, 17.69% for the filtered image, and 6.69% using background correction. Furthermore, the best subtraction index was also obtained by the proposed framework, with a 78.62% in comparison with 11.37% using only the modified histogram-based technique for background correction.

**Fig. 6:**
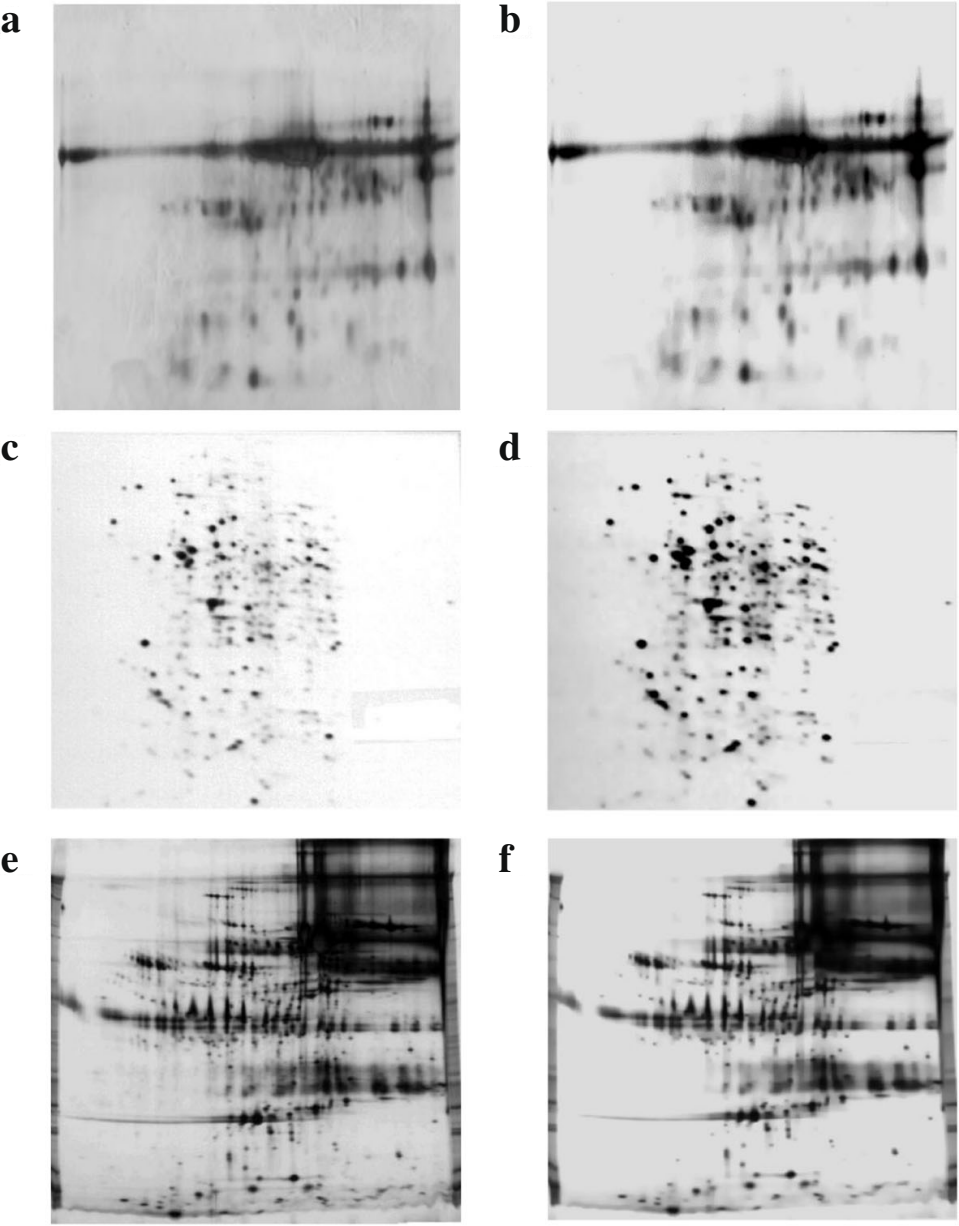
Results of the joint pre-processing framework in real two-dimensional gel electrophoresis – 2-DGE – images. **a** Original 2-DGE image from a human urine sample (samp_05). **b** 2-DGE image of samp_05 with the joint pre-processing framework. **c** Original 2-DGE image from Molt-4 cell line (samp_09). **d** 2-DGE image of samp_09 with joint pre-processing framework. **e** Original 2-DGE image from Fetal Alchohol Syndrome serum (samp_10). **f** 2-DGE image of samp_10 with joint pre-processing framework

Figure 6 presents the effects of the joint pre-processing framework in three of the real 2-DGE images (samp_05–09–10). In the three processed images (Fig. 6b, d, and f), we can see the effect of noise reduction and background homogenization. Additionally, the enhancement of low abundance spots is noticeable.

### Proposal novelties II: validation with real 2-DGE images

The joint pre-processing framework was validated using real 2-DGE images captured from four apitoxin (honey bee venom) samples, two urine samples from patients with prostate cancer, and four 2D images from the LECB 2-D PAGE Gel Image Database. Table 6 presents the number of detected spots from the original and pre-processed samples, as well as the true positives and false positives. We obtained the false positive reduction percentages comparing the original and pre-processed images. For the 2-DGE images of apitoxin (samp_01–02–03–04), the joint pre-processing framework reduced the false positives between 43% and 72%. For the urine samples (samp_05–06), the false positives from the pre-processed images decreased by 91% and 85% respectively. And for the four images from the LECB 2-D PAGE Gel Image Database (samp_07–08–9–10), the false positives were reduced between 71% and 93%. From these results, we can see that the joint pre-processing framework improves protein detection by reducing the false positives caused by noise and non-homogeneous background.

**Table 6:** True positive and false positive spots detected from original and processed real 2-DGE images

	Real image	Number of detected spots	True positives	False positives	False positive reduction
samp_01	Original	1894	34	1860	72%
	Processed	549	34	515	
samp_02	Original	1822	28	1794	43%
	Processed	1043	28	1015	
samp_03	Original	1239	27	1212	49%
	Processed	646	28	618	
samp_04	Original	1698	33	1664	65%
	Processed	611	34	578	
samp_05	Original	1287	248	1039	91%
	Processed	346	248	98	
samp_06	Original	2290	332	1958	85%
	Processed	632	332	300	
samp_07	Original	2115	325	1790	90%
	Processed	504	325	179	
samp_08	Original	2222	253	1969	89%
	Processed	478	253	225	
samp_09	Original	3235	287	2948	93%
	Processed	508	287	221	
samp_10	Original	1795	345	1795	71%
	Processed	771	345	426	

## Conclusions

2-DGE images commonly present several anomalies that hinder spot detection and analysis. In this paper, the use of several digital image processing techniques were tested and validated in three stages, i.e., normalization, noise reduction and background correction, achieving an enhancement of the image for posterior analysis. Each approach helps improve specific anomalies, and here we introduce a new joint pre-processing framework that combines the capabilities of the selected techniques for each of the three stages.

The techniques used in each of the stages of image pre-processing were compared on synthetic images, using four validation measures, i.e., LPD, SNR, spot efficiency (*Ξ*) and BSI, which offered representative and consistent values associated with pre-processing performance, so these quantitative indicators proved to be a very useful measure for 2-DGE image applications.

Experimental results from synthetic images demonstrated that the order of the stages impacts the final results. E.g., if the noise reduction stage is executed before normalization, the faint spots, that have important information for the interpretation of the image, are often removed. Consequently, the order with the best performance was the following: 1) normalization, 2) noise reduction and 3) background correction. In particular, the best normalization technique was adaptive piecewise histogram equalization, according to the LPD validation measure. Equalization techniques enhance the contrast of low-abundance spots, but also increase the background noise. In noise reduction tests, the nonlinear technique GNDF was implemented, which is a new technique for these kinds of images and reduces noise while preserving edges. GNDF showed a similar spot efficiency (*Ξ*) to WT, but a better SNR using synthetic data with different types of noise. Finally, three techniques were compared for background correction using the Background Subtraction Index (BSI). The best results of BSI were obtained using multi-level thresholding.

Results with real 2-DGE images showed that the joint framework outperforms results from a single approach. According to these results, the use of adaptive piecewise histogram equalization, GNDF and multi-level thresholding, is recommended for these kinds of images. However, as future work, the joint pre-processing framework could implement other kinds of techniques for each step, that were not considered in this study.

## Data Availability

The datasets used and/or analyzed during the current study are available from the corresponding author on reasonable request.

## Abbreviations

2-DGE: two-dimensional gel electrophoresis
TV: total variation
WT: wavelet transform
WTTV: Wavelet and total variation
GNDF: geometric nonlinear diffusion filtering
LPD: low-abundance protein detected
SI: subtraction index
SNR: signal-to-noise ratio
